# Use of adaptive traction for endoscopic submucosal dissection when using a bipolar knife for cardiological reasons

**DOI:** 10.1055/a-2253-8963

**Published:** 2024-02-22

**Authors:** Elena De Cristofaro, Louis Jean Masgnaux, Jérôme Rivory, Jérémie Jacques, Jean Grimaldi, Mathieu Pioche

**Affiliations:** 1 Gastroenterology Unit, Department of Systems Medicine, University of Rome Tor Vergata, Rome, Italy; 2 Gastroenterology and Endoscopy Unit, Edouard Herriot Hospital, Hospices Civils de Lyon, Lyon, France; 3 Gastroenterology and Endoscopy Unit, Dupuytren University Hospital, Limoges, France

Endoscopic submucosal dissection (ESD) allows en bloc resection of gastrointestinal lesions, providing completeness of resection and facilitating accurate histological assessment. The knives currently in use for ESD are monopolar devices that can cause electromagnetic interference with cardiac implantable electronic devices (CIEDs). Speedboat (Speedboat-RS2; Creo Medical Ltd, Chepstow, UK) is a novel bipolar device designed for ESD, which utilizes advanced energy (radiofrequency and microwave) for dissection and coagulation. It is an all-in-one device, capable of executing submucosal injection, dissection, and coagulation, making the procedure faster without interfering with CIEDs 1 . We report the use of this device during ESD with an adaptive traction instrument (A-TRACT 2+2; Hospices civils de Lyon, Lyon, France), the benefits of which have been described previously ( Video 1 ) 2 3 4 5 .

Use of the adaptive traction device combined with a bipolar knife for endoscopic submucosal dissection of a gastric lesion.Video 1

A 75-year-old patient was referred to our center with a gastric flat lesion, 30×35 mm in size (high grade dysplasia at previous biopsies). ESD was indicated and a bipolar knife was preferred because of the patient’s pacemaker. After circumferential incision and trimming, we placed the A-TRACT 2+2 by fixing the two loops on lateral edges of the lesion and the rubber band to the opposite wall ( Fig. 1 ). The dissection was started with appropriate traction and good submucosal exposure. After cutting half of the lesion, the adaptive traction was tightened to re-establish proper traction. The Speedboat device allowed safe dissection, and effective and immediate treatment of intraprocedural bleeding, without requiring any cardiological precaution due to the presence of the pacemaker. In addition, the traction device facilitated the procedure by ensuring optimal submucosal exposure until the end of the procedure. The procedure was completed in 40 minutes without events adverse, and the histopathology revealed curative resection of high grade dysplasia.

**Fig. 1:**
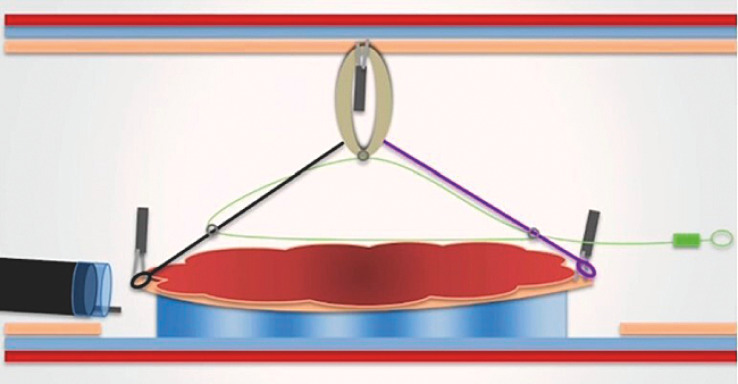
Schematic representation of the adaptive traction device (A-TRACT 2+2; Hospices civils de Lyon, Lyon, France).

We suggest that the adaptative traction strategy could facilitate ESD, even when this new bipolar knife is selected in order to avoid complications in patients with CIEDs.

Endoscopy_UCTN_Code_TTT_1AO_2AG

## Notes

Endoscopy E-Videos https://eref.thieme.de/e-videos
*E-Videos* is an open access online section of the journal *Endoscopy* , reporting on interesting cases and new techniques in gastroenterological endoscopy. All papers include a high-quality video and are published with a Creative Commons CC-BY license. Endoscopy E-Videos qualify for HINARI discounts and waivers and eligibility is automatically checked during the submission process. We grant 100% waivers to articles whose corresponding authors are based in Group A countries and 50% waivers to those who are based in Group B countries as classified by Research4Life (see: https://www.research4life.org/access/eligibility/ ). This section has its own submission website at https://mc.manuscriptcentral.com/e-videos .
